# Jugular bulb anatomical variations and pneumatization patterns: a comprehensive CBCT analysis

**DOI:** 10.1007/s00276-024-03401-1

**Published:** 2024-06-07

**Authors:** Răzvan Costin Tudose, Mugurel Constantin Rusu, George Triantafyllou, Maria Piagkou, Liliana Moraru, Cătălin Constantin Dumitru

**Affiliations:** 1 Division of Anatomy, Faculty of Dentistry, “Carol Davila” University of Medicine and Pharmacy, Bucharest, 020021 Romania; 2 Department of Anatomy, School of Medicine, Faculty of Health Sciences, National and Kapodistrian University of Athens, Athens, 11527 Greece; 3 Department of Oral and Maxillofacial Surgery, “Carol Davila” Central Military Emergency Hospital, Bucharest, Romania; 4 Faculty of Dentistry, “Titu Maiorescu” University, Bucharest, Romania; 5 Davila” University of Medicine and Pharmacy, 8 Eroilor Sanitari Blvd, Bucharest, RO-050474 Romania

**Keywords:** Jugular bulb, Pneumatization patterns, Anatomical variation, Internal acoustic canal, Infralabyrinthine, Skull base surgery

## Abstract

**Purpose:**

This study aims to assess the anatomical possibilities of the jugular bulb (JB).

**Methods:**

Fifty archived CBCT scans were analyzed.

**Results:**

The average distance between the internal acoustic canal (IAC) and the JB was 7.97 mm on both sides (Right: SD = 2.56 mm, range 3.16–13.3 mm; Left: SD = 2.5 mm, range 2.9–13.6 mm). JB walls’ pneumatization was classified into eight patterns. Deep petrosal cells (DPCs) prevailed in the lateral wall of the JB. The absence of pneumatization (NP) was commonly found on the left side. The presence of infralabyrinthine and hypotympanic cells varied. Less common types included accessory occipital cells (AOCs), posteromedial tracts (PMTs), and basi-occipital cells (BOCs), which determined a consistent variation of the lateral wall pneumatization patterns. Pneumatization of the medial wall was not observed in 50 right sides and 49 left sides. The inferior wall analysis revealed symmetry in AOC distribution and a predominant occurrence of NP. Cases with hypotympanum (HT) in the lateral wall showed a statistically significant IAC-JB distance increase by an average of 4.67 mm compared to NPs. Specific pneumatizations, particularly HT on the lateral side, have a significant effect on the IAC-JB distance, showing a clear pattern of increasing distance from DPC to NP and then to HT. A significant distance increase in HT pneumatization was noted. There were also recorded instances of JB hypoplasia and hyperplasia, JB diverticula, dehiscent JBs, and high JBs.

**Conclusion:**

This study establishes a novel classification of JB pneumatizations to aid in the understanding of the temporal bone anatomy.

## Introduction

The jugular bulb (JB) is an essential structure in the cranial venous system, bridging the sigmoid sinus with the internal jugular vein within the depths of the jugular fossa and lying posterolaterally in the pars vascularis region of the jugular foramen [13]. The juxtaposition of the JB with cranial nerves IX, X, and XI, alongside its connection to the cavernous sinus through the inferior petrosal sinus, underlines the delicate interplay between the venous channels and neural pathways at the skull base [5]. The JB is anatomically localized at the floor of the middle ear cavity near the labyrinth and the mastoid segment of the facial nerve, being closely associated with auditory functions [27]. Typically, the upper boundary of the JB is situated beneath the hypotympanum, positioning it at a critical juncture near several key anatomical features of the temporal bone [28].

The jugular foramen is relevant for lateral skull base surgery, particularly when accessing extracranial or intracranial diseases [24]. The accessibility of the JB is critical in surgeries not directly involving it, such as neurotologic [29] or cardiac [36] surgery. This is necessary to accurately identify and manage the normal venous drainage pathways from the lateral skull or the infratemporal fossa [5]. It sets the boundary for exposure in various surgical approaches, such as retrolabyrinthine, translabyrinthine, and transcochlear, directed toward the posterior fossa [15]. The exposure techniques and manipulation of the JB, typically limited to skeletonization and cauterization, underscore the necessity for precise anatomical understanding and cautious surgical planning to preserve the integrity of this complex region [26].

While numerous studies have investigated pneumatizations of the temporal bone [2, 9, 33], or anatomical variations of the JB [20, 45] and adjacent veins [19, 34], research specifically employing cone beam computed tomography (CBCT) to thoroughly assess and classify these features around the JB and their impact on its positioning has been limited. Our study seeks to bridge this gap in the literature. To our knowledge, not only are we pioneering the use of CBCT for an in-depth examination of JB pneumatizations, but we are also the first to classify these pneumatizations into eight distinct types. This dual focus on advanced imaging technology and detailed classification aims to provide a more nuanced understanding of how pneumatizations influence the anatomical placement of the JB, offering valuable insights that could inform future medical and surgical approaches.

## Materials and methods

In this retrospective study, detailed anatomical assessments and measurements were conducted on 50 anonymized adult CBCT scans, comprising 35 females and 15 males, which equated to 100 analyzable sides. The cases were explored for dental medical purposes.

Prior to data acquisition, all scans were carefully oriented to guarantee measurement precision. The scans were performed using a high-resolution iCat CBCT machine (Imaging Sciences International, Hatfield, PA, USA), with the parameters set to a resolution of 0.250, a field of view of 130, and an image matrix of 640 × 640. Uncompressed DICOM files were the format for CBCT file exportation and were subsequently analyzed utilizing the Planmeca Romexis Viewer 3.5.0.R software, consistent with methodologies applied in prior studies [16, 32]. The multiplanar reconstructions (MPRs) emphasized the coronal and axial anatomical planes, with the sagittal plane referenced for additional detail on the variations as required. To maintain accuracy, given that CBCT can only detect bony structures, we delineated the JB as the osseous anatomical compartment housing the JB, which could be identified on CBCT scans. All participating patients provided written consent for their radiological data to be utilized for research and educational objectives, under the stipulation that their identity and personal information remain confidential. The research was conducted ethically in accordance with the Code of Ethics of the World Medical Association (Declaration of Helsinki), and the study was approved by the responsible authorities (ethical agreement no.437/2021—affiliation 3).

To ensure accuracy and reliability in our study, we combined a third-reviewer evaluation method with Cohen’s kappa analysis. The third reviewer resolved discrepancies between initial evaluations, ensuring precision on a case-by-case basis. We employed Cohen’s kappa (κ) to assess the consistency and reliability of the initial reviewers’ agreement, seeking a statistical measure of their evaluative alignment for the categorical variables. The threshold for Cohen’s kappa was set at 0.60, aiming to capture substantial to almost perfect agreement between reviewers. This dual methodology strengthened both individual assessment accuracy and the overall integrity of our findings, affirming the significant level of inter-rater agreement necessary for our research.

### Internal acoustic canal to jugular bulb distance and positioning

Measurements focused on the distances between the internal acoustic canal (IAC) and the JB, evaluated both coronally—from the apex of the JB to the IAC’s floor—and axially, noting the IAC’s position relative to the JB, categorized as neutral (directly superior), slightly anterior (2.5 to 5 mm distance between IAC and JB), or anterior (over 5 mm distance between IAC and JB).

A quartile-based classification method was used to divide the set of values into four sets of values. Firstly, the data points were ordered from smallest to largest, after which the values were segmented into quartiles through the identification of three key percentile values: the first quartile (Q1), marking the 25th percentile, the second quartile (Q2), or median, at the 50th percentile, and the third quartile (Q3), at the 75th percentile. These quartile thresholds served as demarcation points that separated the dataset into four equal parts.

The axial plane distance between the JB and IAC was measured and classified into three categories: 0–2.5 mm as neutral (JB and IAC in the same coronal plane), 2.5–5 mm as slightly anterior (JB slightly anterior to IAC), and > 5 mm as anterior (JB significantly anterior to the IAC).

### Pneumatization types

**Fig. 1:**
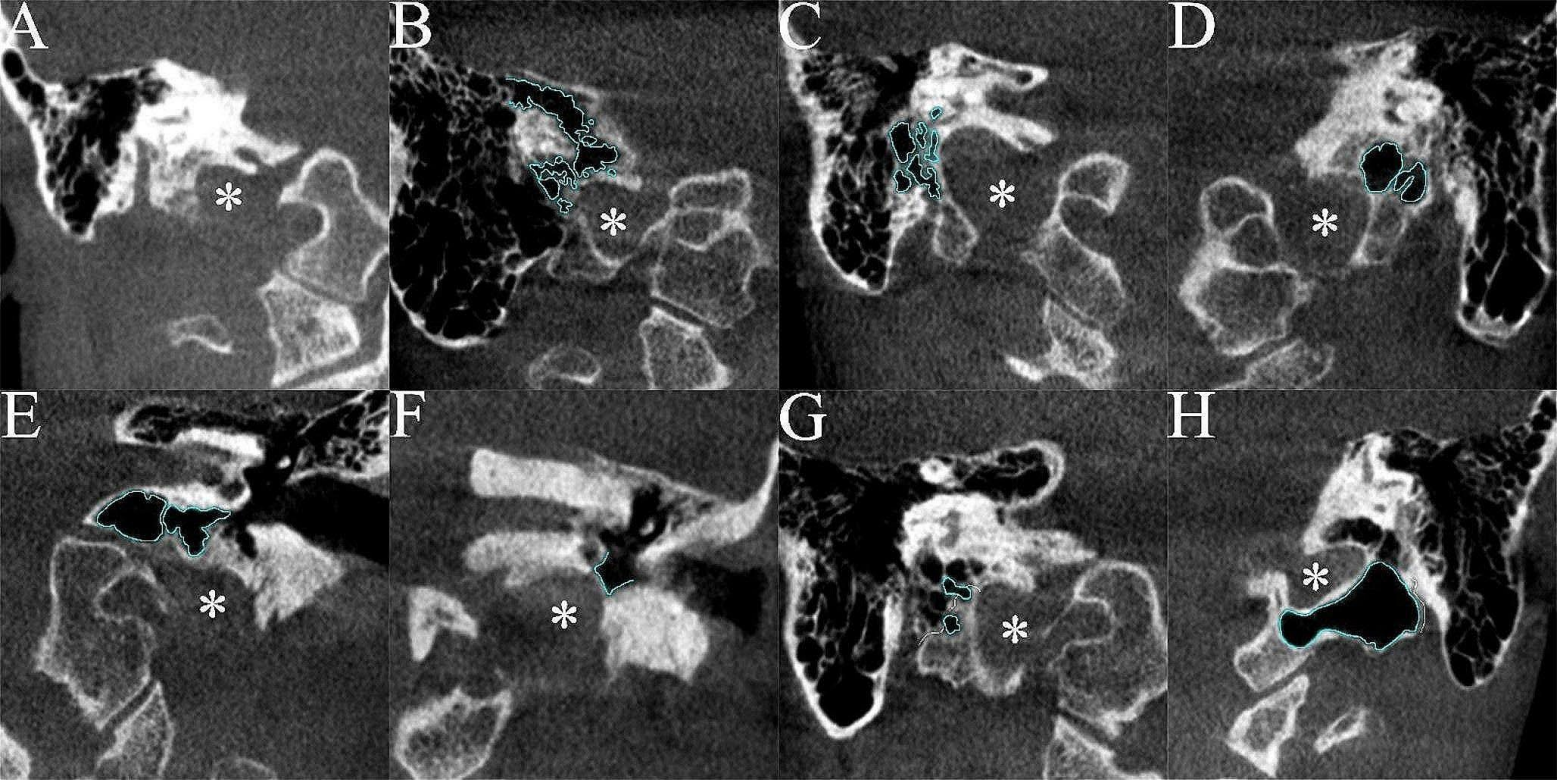
Detailed coronal views of the jugular bulb (*) pneumatization variants. Each panel, labeled A to H, represents a distinct type of pneumatization observed within the study population. Surrounding air cells are delineated with a blue outline for enhanced visibility

Pneumatization within the perilabyrinthine area was systematically classified according to a version of Allam et al.’s classification system (Table 1) [2], specifically adapted for the region surrounding the JB. This analysis focused on the JB’s walls but did not include the anterior and posterior ones. The evaluation thoroughly examined the superior, inferior, lateral, and medial walls of the JB, carefully assessing these areas for different patterns of pneumatization. Eight distinctive types of pneumatization were identified, as detailed in Table 2; Fig. 1.

**Table 1:** Classification of the temporal bone pneumatization, according to Allam et al. (1969)

Region	Area	Tract
A. Mastoid	1. Antrum	Aditus
	2. Periantral	Antral
	3. Tegmental	Antral
	4. Sinodural	Antral
	5. Perisinus	Antral
	6. Central	Antral
	7. Mastoid tip (lateral/medial)	Antral
	8. Perifacial	Hypotympanic
B. Perilabyrinthine	1. Supralabyrinthine	Posterosuperior
		Posteromedial
		Subarcuate
	2. Infralabyrinthine	Hypotympanic
		Retrofacial
C. Petrous Apex	1. Peritubal	Anterosuperior
		Anterolateral
	2. Apical	Hypotympanic
		Peritubal
		Perilabyrinthine
D. Accessory	1. Zygomatic	
	2. Squamous	
	3. Occipital	
	4. Styloid	

**Table 2:** A concise overview of various types of pneumatization found in the vicinity of the jugular bulb

Pneumatization Type	Abbreviation	Description	Figure	
Singular pneumatizations				
No pneumatization	NP	Defined by the absence of pneumatization within a 5 mm radius of the JB.	1 A	
Posteromedial tract	PMT	A tract within the supralabyrinthine area that extends from the mastoid along the posteromedial surface of the petrous bone.	1B	
Deep petrosal cell	DPC	Originating from the deep portion of the antrum, these cells form from a common tract or individual channels, descending posteriorly and parallel to the facial canal, contributing to the deep cells of the mastoid process.	1 C	
Infralabyrinthine cell	ILC	Cells that arise from the most inferior part of the antrum, projecting downward and inward beneath the posterior semicircular canal and near the JB, with denser septa compared to DPCs.	1D	
Hypotympanic cell	HTC	Cells that extend medially from the hypotympanum towards the region of the JB, situated inferiorly to the labyrinth.	1E	
Hypotympanum	HT	Referring to the area of the hypotympanum.	1 F	
Basi-occipital cell	BOC	A group of cells that may extend through a fused petro-occipital suture, passing beneath the terminal portion of the sigmoid sinus into the basilar part of the occipital bone.	1G	
Accessory occipital cell	AOC	A significant cell within the occipital bone sharing the same origin as the BOCs.	1 H	
Conjoined pneumatizations				
Deep petrosal cell – hypotympanic cell	DPC-HTC	Two various air cells conjoined and they could not be distinctly classified as a single type		-
Deep petrosal cell – infralabyrinthine cell	DPC-ILC			-
Hypotympanic cell – infralabyrinthine cell	HTC-ILC			-

**Fig. 2:**
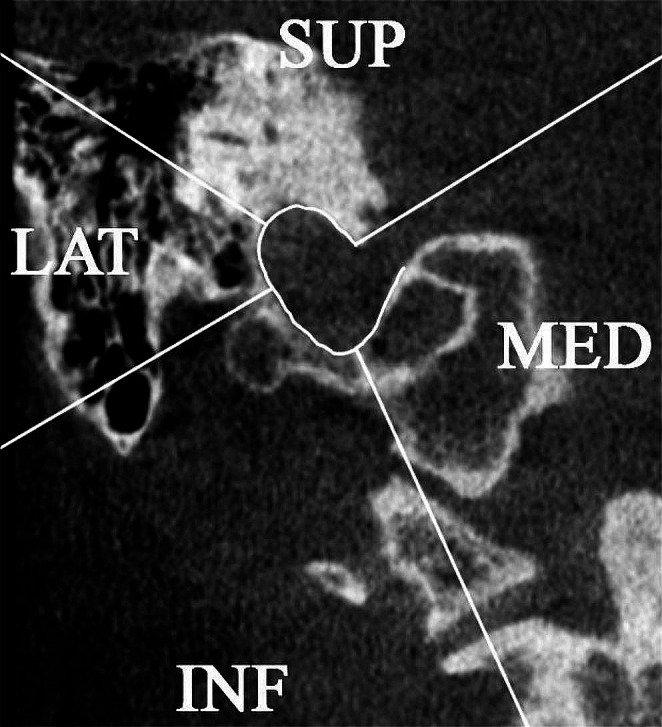
Detailed coronal view, highlighting the jugular bulb (outlined in white). The illustration clearly demarcates the sides—superior (SUP), inferior (INF), lateral (LAT), and medial (MED)—to which different types of pneumatization are attributed

Each type of pneumatization was attributed to a side (superior, inferior, lateral and medial) as seen in Fig. 2. Inferior and medial pneumatization types were attributed if the air cells were situated in the occipital bone, while the superior and lateral pneumatization were located in the temporal bone.

### Anatomical variations of the jugular bulb

**Fig. 3:**
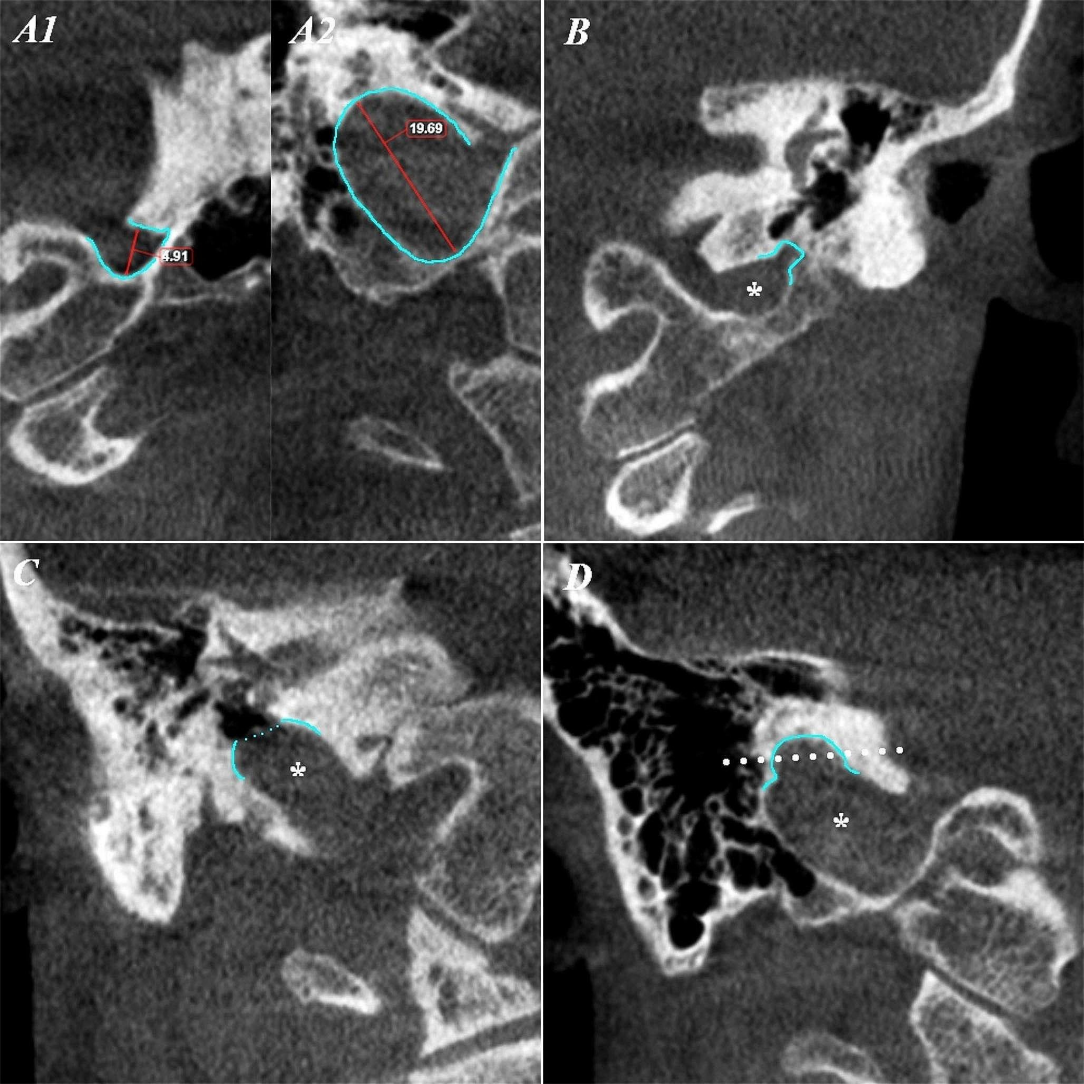
Detailed coronal views of the jugular bulb (*) anatomical variations. Each panel represents an anatomical variation analyzed in this study. A1: hypoplasia; A2: hyperplasia; B: diverticulum; C: dehiscence of the jugular bulb; D: high jugular bulb (the level of the floor of the internal acoustic canal was outlined with the white dotted line)

Bilateral anatomical variations of the JB were explored, including aspects such as hypoplasia, hyperplasia, dehiscence, and the presence of JB diverticula (JBD), along with instances of high JBs (HJBs). Hypoplasia was defined in cases where the JB’s maximum diameter was 5 mm or less, while hyperplasia was indicated by a JB with a minimum diameter of 15 mm, due to the lack of reports in literature on size thresholds. The JBD was characterized as an extraluminal outpouching from the JB [11], identifiable in planar sections centered on the JB, denoting any protrusion that significantly alters the typical morphology of the JB. A HJB was classified as reaching the level of the IAC floor or ascending above it [43]. Dehiscence referred to instances where the bony wall between the JB and the middle ear (the jugular plate) was absent [6]. (Fig. 3)

### Statistical analysis

Our analysis commenced with Excel Descriptive Statistics to overview our subgroups, focusing on key statistical metrics. Subsequently, we employed Jamovi software (version 2.3.21.0) and R software (version 4.1.2) for detailed investigations [41, 42], using one-way ANOVA and Games-Howell post-hoc tests to examine the impact of pneumatization types on IAC-JB distances. This approach allowed for both an initial assessment and an in-depth analysis of our data.

We used a quantitative approach to determine the relationship between the distance from the IAC to the JB and various pneumatization types within the superior and lateral walls. The IAC-JB distance was used as a continuous variable, recorded to two decimal places. The pneumatization types, identified as nominal categories, served as our grouping variable. Statistical analysis was conducted via a one-way ANOVA, specifically using Welch’s test to account for the assumption of unequal variances across groups. Post-hoc comparisons were facilitated through the Games-Howell test, which is appropriate for datasets with unequal variances and different sample sizes. This test provided us with the mean differences between groups and assessed the significance of these differences, enabling a comprehensive understanding of the impact of pneumatization type on the IAC-JB distance. Results were reported with mean differences and associated p-values to signify statistical significance, with a conventional alpha level of 0.05. Exclusion criteria for pneumatization types were set to enhance the robustness of the analysis. Each pneumatization type was required to appear in at least two instances within a given wall to be considered for the one-way ANOVA. Consequently, for the lateral wall, 96 cases were included, while 4 cases featuring singular occurrences of BOC, AOC, PMT, and DPC-HTC were excluded due to insufficient data points. Similarly, for the superior wall, the analysis encompassed 98 cases, with 2 exclusions where the combinations HTC-ILC and DPC-ILC appeared only once. Regarding the inferior wall, the analysis was conducted with 98 cases, after excluding one case each of ILC and BOC due to their solitary presentations in the dataset. The analysis for the medial wall pneumatization was not feasible due to the dataset containing 99 instances of NP and only one case of AOC, precluding statistical testing. This selection criteria for data inclusion was necessary to fulfill the software requirements for a valid ANOVA process and to maintain the integrity of our statistical analysis.

## Results

### Internal acoustic canal to jugular bulb distance and positioning

The IAC-JB distance measurements on both the right and left sides exhibited symmetrical distribution with identical means of 7.97 mm. The standard deviations (SDs) were slightly different, with the right side having an SD of 2.56 and the left side a marginally lower SD of 2.50, indicating a slightly tighter clustering of values around the mean on the left (Table 3).

**Table 3:** Quartile distribution of distances between the internal acoustic canal and the jugular bulb

Metric	Right Side	Left Side
Mean	7.97	7.97
Minimum	3.16	2.9
Maximum	13.3	13.6
Standard Deviation	2.56	2.50
Q1 Range	**≤ 5.62**	**≤ 6.13**
Q1 Count	12	13
Q2 Range	**> 5.62 to ≤ 8.02**	**> 6.13 to ≤ 7.83**
Q2 Count	12	12
Q3 Range	**> 8.02 to ≤ 9.75**	**> 7.83 to ≤ 9.41**
Q3 Count	13	12
Q4 Range	**> 9.75**	**> 9.41**
Q4 Count	12	12

The quartile distribution on the right side demonstrated that the minimum and maximum values spanned a broader range, from 3.16 to 13.3 mm, compared to the left side’s range of 2.9 to 13.6 mm. This is interesting considering both sides have the same mean, suggesting that while the central tendency was similar, the spread and extreme values differed slightly.

For the right side, the first quartile (Q1) included values up to and including 5.62 mm, with a count of 12 values, indicating that a quarter of the measurements were in the lower segment of the dataset. The second quartile (Q2) contained values greater than 5.62 mm but up to and including 8.02 mm, also with 12 values, marking the lower-middle range. The third quartile (Q3) surpassed Q2 and included values up to and including 9.75 mm, comprising 13 values and indicating the upper-middle range. Finally, the fourth quartile (Q4) included all values greater than 9.75 mm, again with 12 values, signifying the highest quarter of measurements.

The left side displayed a similar pattern, with the first quartile (Q1) including values up to and including 6.13 mm, with a count of 13 values. The second quartile (Q2) ranged above 6.13 to 7.83 mm, with 12 values. The third quartile (Q3), showing values greater than 7.83 mm up to and including 9.41 mm, also included 12 values. The fourth quartile (Q4) held all values greater than 9.41 mm, with 12 values present.

Two instances of an HJB, one on each side, were identified where the JB ascended above the floor of the IAC, resulting in negative IAC-JB distances: -1.75 mm on the right and − 1.25 mm on the left. These cases were excluded from the previous analysis to maintain the integrity of the results.

In axial plane positioning, the right side revealed 26 instances with the IAC neutrally positioned relative to the JB, 21 slightly anterior, and 3 distinctly anterior, with a distance greater than 5 mm. On the left side, there were 30 neutral positions, 18 slightly anterior, and 2 anterior cases.

### Pneumatization patterns

The Cohen’s Kappa test coefficient was 0.917, showing an almost perfect agreement between reviewers. In examining the JB’s lateral wall pneumatization, notable findings included a predominant presence of DPCs, with 25 cases on the right and 23 on the left. NPs were more frequent on the left (11) compared to the right (6). HTCs and ILCs also showed variations, with HTCs found in 9 cases on the right and 7 on the left, while ILCs were observed in 2 cases on the right and 6 on the left. Other types such as AOCs, PMTs, and BOCs were less common or absent, indicating a significant discrepancy in pneumatization patterns of the lateral wall (Table 4).

**Table 4:** Distribution of pneumatization patterns across jugular bulb walls

Area	Pneumatization type	Right		Left	
		**No. of cases**	**Prevalence (%)**	**No. of cases**	**Prevalence (%)**
Lateral					
	DPC	25	50	23	46
	NP	6	12	11	22
	AOC	1	2	0	0
	PMT	1	2	0	0
	HTC	9	18	7	14
	ILC	2	4	6	12
	BOC	1	2	0	0
	HT	4	8	3	6
	DPC-HTC	1	2	0	0
Medial					
	NP	50	100	49	98
	AOC	0	0	1	2
Superior					
	DPC	4	8	4	8
	NP	11	22	9	18
	AOC	0	0	0	0
	PMT	6	12	2	4
	HTC	23	46	19	38
	ILC	1	2	6	12
	HT	1	2	7	14
	DPC-HTC	3	6	2	4
	DPC-ILC	1	2	0	0
	HTC-ILC	0	0	1	2
Inferior					
	AOC	6	12	6	12
	NP	42	84	44	88
	BOC	1	2	0	0
	ILC	1	2	0	0

A singular case on the right side featuring a combination of DPC and HTC (DPC-HTC) was excluded from the initial analysis, as it didn’t align with a singular category of pneumatization.

For the JB’s medial wall, results showed NP in all 50 cases on the right and 49 on the left, with a unique occurrence of an AOC on the left (Table 4).

HTCs emerged as the most frequent finding on the JB’s superior wall, with 23 occurrences on the right and 19 on the left. NPs were also significant, particularly on the right (11) compared to the left (9). DPCs presented evenly on both sides, 4 each, while the left side had a notable quantity of ILCs (6) and HTs (7), surpassing the right’s count of ILC (1) and HT (1). PMTs were more observed on the right (6) than on the left (2), with AOCs and BOCs absent on both sides.

In the superior wall analysis, a higher frequency of combined cell occurrences compared to other walls was noted, with 7 cases of conjoined pneumatizations. Specifically, there was a pairing of a DPC and an ILC (DPC-ILC) in one instance, a more frequent combination of DPCs and HTCs (DPC-HTC) in five cases, and a single occurrence of an HTC and an ILC (HTC-ILC) together. This prevalence of combined cells highlights a distinctive complexity within the superior wall’s pneumatization patterns (Table 4).

Within the inferior wall of the JB, the analysis highlighted a symmetry in AOCs with an equal distribution of 6 on both the right and left sides. A notable prevalence of NPs was observed, accounting for 42 cases on the right and 44 on the left. The presence of BOCs was exclusive to the right with a single instance, and ILCs were similarly singular on the right, absent on the left, suggesting side-specific variations in pneumatization patterns (Table 4).

### Impact of pneumatization types on IAC-JB distance variability

In analyzing the lateral aspect of the JB, the Games-Howell post-hoc test revealed notable findings regarding the distances between the IAC and the JB. Cases with HT on the lateral side showed an average increase in the IAC-JB distance of 4.67 mm over those with NP, a statistically significant difference (*p* = .015). This suggests that in the presence of HT pneumatization laterally, the JB is positioned further from the IAC by this measured amount on average. Additionally, while not reaching statistical significance, the comparison of DPC to HT on the lateral side indicated a trend with HT cases having an increased mean distance of 3.67 mm (*p* = .054). The lack of significant differences in IAC-JB distances across other types of pneumatization on the lateral side emphasizes the specific impact of HT in altering JB positioning relative to the IAC (Table 5).

**Table 5:** Games-Howell post-hoc comparisons of distances between the internal acoustic canal (IAC) and the jugular bulb (JB) across different pneumatization types in the lateral wall is displayed. It lists mean differences and their statistical significance (p-values), revealing notable variations such as between NP and HT types, indicating how pneumatization affects IAC-JB distance

Pneumatization Type		DPC	NP	HTC	ILC	HT
DPC	Mean difference	—	-1.00	0.909	-0.07	3.67
	p-value	—	*p* = .669	*p* = .619	*p* = 1.000	*p* = .054
NP	Mean difference	—	—	1.912	0.937	4.67
	p-value	—	—	*p* = .112	*p* = .833	*p* = .015
HTC	Mean difference	—	—	—	-0.98	2.76
	p-value	—	—	—	*p* = .752	*p* = .175
ILC	Mean difference	—	—	—	—	3.74
	p-value	—	—	—	—	*p* = .063
HT	Mean difference	—	—	—	—	—
	p-value	—	—	—	—	—

In assessing the superior wall of the JB, the Games-Howell post-hoc test emphasized several statistically significant differences in the distances between the IAC and the JB when comparing pneumatization types. A particularly noteworthy finding was the significant mean increase in the IAC-JB distance of 5.765 mm in cases with HT compared to DPC, with a highly significant p-value of less than 0.001. Conversely, when comparing NP to DPC, there was a mean decrease in distance of 4.82 mm, indicating that DPC is associated with a shorter distance compared to NP, also significant with a p-value of 0.002. The analyses reveal that the type of pneumatization present can significantly affect the IAC-JB distance, which has potential clinical implications for understanding anatomical variation in the superior wall of the JB region (Table 6).

**Table 6:** Games-Howell post-hoc test examines the differences in distances between the internal acoustic canal and the jugular bulb across various types of pneumatization within the superior wall. It details the mean differences between each pneumatization type, with negative values indicating a smaller average distance for the row group compared to the column group. The accompanying p-values assess the statistical significance of these differences, with values below 0.05 typically considered significant, suggesting that specific pneumatization patterns may substantially affect the spatial relationship in this region of the skull

Pneumatization Type		NP	DPC	HTC	ILC	HT	PMT	DPC-HTC
NP	Mean Difference	—	-4.82	-1.71	-3.62	0.944	-0.237	-4.479
	p-value	—	*p* = .002	*p* = .255	*p* = .012	*p* = .932	*p* = 1.000	*p* = .001
DPC	Mean Difference	—	—	3.11	1.20	5.765	4.584	0.341
	p-value	—	—	*p* = .039	*p* = .872	*p* < .001	*p* = .186	*p* = 1.000
HTC	Mean Difference	—	—	—	-1.91	2.652	1.470	-2.772
	p-value	—	—	—	*p* = .186	*p* = .025	*p* = .950	*p* = .031
ILC	Mean Difference	—	—	—	—	4.565	3.384	-0.859
	p-value	—	—	—	—	*p* = .002	*p* = .436	*p* = .934
HT	Mean Difference	—	—	—	—	—	-1.181	-5.424
	p-value	—	—	—	—	—	*p* = .987	*p* < .001
PMT	Mean Difference	—	—	—	—	—	—	-4.242
	p-value	—	—	—	—	—	—	*p* = .221
DPC-HTC	Mean Difference	—	—	—	—	—	—	—
	p-value	—	—	—	—	—	—	—

In the examination of the inferior wall pneumatization of the JB, the Games-Howell post-hoc test compared the NP group to the AOC group, which were the only pneumatization types meeting the eligibility criteria for this comparison. The analysis revealed a mean difference of 1.71 mm, with the AOC group showing a tendency towards an increased distance from the IAC compared to the NP group. However, this difference did not reach statistical significance, as reflected by a p-value of 0.117. This suggests that while there is a numerical difference in IAC-JB distance between the two pneumatization types on the inferior wall, it is not sufficient to assume a definitive impact of AOC pneumatization on the anatomical layout within this region (Table 7).

**Table 7:** Games-Howell post-hoc statistical test results comparing two types of pneumatization — NP and AOC in the inferior wall. It presents the mean difference between these groups along with the associated p-values to evaluate statistical significance

Pneumatization Type		NP	AOC
NP	Mean Difference	—	1.71
	p-value	—	*p* = .117
AOC	Mean Difference	—	—
	p-value	—	—

### Anatomical variations of the jugular bulb

**Fig. 4:**
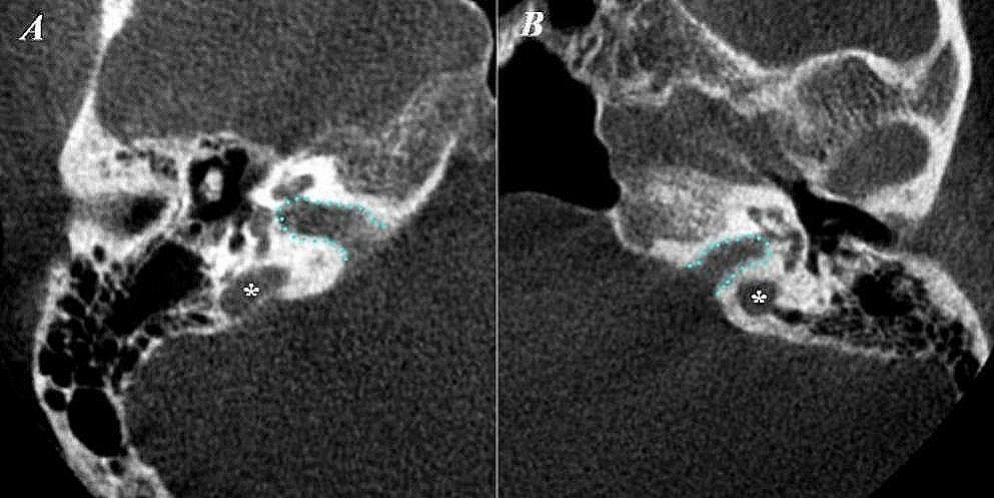
Axial view of the high jugular bulbs (*) and the internal acoustic canal (outlined with blue)

Our examination of the JB revealed distinct anatomical variations (Fig. 3): hypoplasia in 7 instances (A1), hyperplasia in 10 (A2), and JBD in 8 cases (B). Notably, we encountered 2 instances of both dehiscent JB (C) and HJB (D)(Fig. 4). The evaluation covered both the right and left sides, with a total sample size of 100 cases. Therefore, the prevalence of each feature corresponds to the number of cases in which it was observed. (Table 8)

**Table 8:** Prevalence of the main anatomical variants of the jugular bulb

Anatomical variant	Prevalence (%)
Hypoplasia	7
Hyperplasia	10
JB Diverticula	8
Dehiscent JB	2
High JB	2

**Fig. 5:**
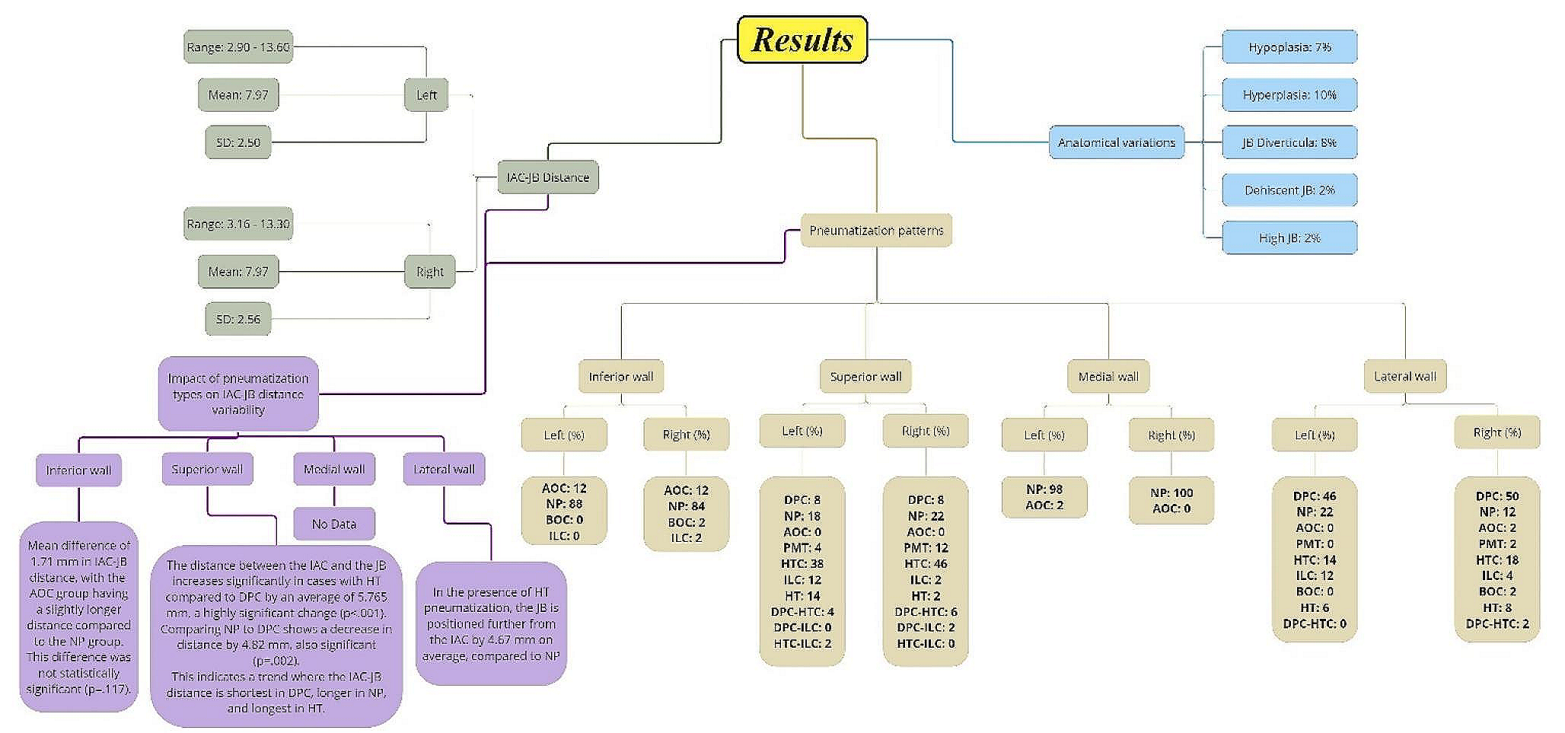
Illustrative representation of the reported study findings

A schematic overview of the results is presented in Fig. 5.

## Discussion

### The distance between the internal acoustic canal and the jugular bulb

Research shows that there are significant variances in anatomical distances between the IAC and the JB. Singla et al. [38] reported a mean distance of 7.18 mm, slightly less than Kolagi et al. [22], which presented a mean of 6.21 mm. Vachata et al. [43] noted a more significant variability, with the mean average being set at 7.5 mm and the range between 1 and 16 mm. Their comparison between cadaveric measurements (mean 7.9 mm) and high-resolution computed tomography (HRCT) measurements (mean 7.1 mm) highlights the potential for imaging techniques to offer a slightly reduced distance due to precise imaging cross-sections, an important consideration for pre-surgical evaluations. Koval et al. [23] observed an average of 6.3 mm on dry skulls, and Roland et al. [31] reported a lower average distance of 4.1 mm. This variability presents significant implications for surgical risk and technique, suggesting that some patients may exhibit anatomical configurations that pose additional challenges for surgical access and JB management. Aslan et al. [3] focused on the height of HJBs with a mean of 9.4 mm. In Gupta et al.‘s study conducted on dry skulls, the shortest distance averaged 7.94 mm on the right and 8.47 mm on the left, with a minimum of about 5 mm across specimens [14]. Advanced imaging, such as CBCT, provides much detail and dynamic three-dimensional views compared to dry skulls, crucial for pre-surgery briefing and studying conditions like Meniere’s Disease. Our findings, with an average distance of 7.97 mm between IAC and JB, align with previous studies and underscore the importance of comparing such measurements with live imaging data for clinical-radiologic relevance.

### Pneumatization in bones surrounding the jugular bulb

In the assessment of temporal bone pneumatization, various classifications have been proposed to categorize this anatomical feature, each highlighting different aspects of pneumatization patterns. Allam’s classification offers a fundamental perspective by delineating pneumatization into Mastoid, Petrous, and Accessory types, focusing on spatial distribution within the temporal bone, a categorization crucial for surgical considerations and radiological evaluations [2]. On the other hand, Singh et al.‘s review discusses existing classifications through a broad lens, emphasizing the variability across five specific regions: middle ear, squamomastoid, perilabyrinthine, petrous apex, and accessory areas [37]. This underlines the importance of understanding the gradation of pneumatization based on cell density and extent, which bears implications for otologic interventions and diagnostic precision. Dexian Tan et al.‘s study contributes to the field by identifying prevalent patterns of pneumatization within distinct compartments of the temporal bone, utilizing HRCT to inform clinical and surgical strategies [9]. While not specifying explicit types, it underscores the practical relevance of recognizing these pneumatization patterns. In contrast, the classifications proposed by Jadhav et al. [17] and Azuma et al. [4] offer a deeper understanding of the quantitative and morphological characteristics of pneumatization. Jadhav et al. utilize CBCT for volumetric analysis, classifying pneumatization based on reference structures. Azuma et al. introduce a morphological classification, delineating five types based on the volume and directional growth of air cells, offering insights into the developmental dynamics of pneumatization. However, despite the knowledge these classifications offer about temporal bone pneumatization, none specifically address the pneumatization patterns around the JB, an area of significant clinical interest due to its implications for surgical access [8, 25] and risk management in otologic and neurotologic procedures [10, 29]. This gap in the literature highlights the need for a specialized approach to understand and classify pneumatization in this critical region. While appreciating the contributions of the existing classifications, we recognized the necessity to focus more closely on the pneumatization around the JB. To this end, we adopted the classification system proposed by Allam et al. [2] as a starting point and adapted it to specifically address the pneumatization patterns around the JB. This adaptation aims to fill the identified gap and provide a more comprehensive understanding of temporal bone pneumatization, enhancing surgical planning and diagnostic precision concerning the JB. The presence of HTCs in the superior wall of the JB has been previously acknowledged, without any prevalence or measurement [12]. For preoperative planning and surgical operations in this area, it is essential to comprehend the particular pneumatization patterns surrounding the JB. Surgeons can reduce the risk of complications by anticipating anatomical variations when these patterns are accurately identified by preoperative imaging.

### Positioning of the jugular bulb relative to temporal bone pneumatization

The debate on how temporal bone pneumatization influences the height of the JB remains unsettled, with varying hypotheses presented in the literature. Graham [12], supported by Wadin et al. [44] and Kennedy et al. [21], posited a connection between HJB and the degree of temporal bone pneumatization, suggesting that HJB is frequently associated with lesser pneumatization levels. In contrast, Aladeyelu et al. [1] found HJB across various pneumatization degrees, notably more prevalent in cases of hyper-pneumatized temporal bone, indicating a significant pattern at *p* < .001. This diverges from Chen et al.‘s findings [7], which aligned with Orr et al. [30] in noting no marked difference in mastoid air cell volume between severe and mild HJB cases, highlighting no direct link between JB positioning and pneumatization extent. However, Orr et al. observed a reliable correlation between certain anatomical distances around the JB, suggesting some degree of spatial consistency regardless of pneumatization. Our research contributes to this complex narrative by identifying the specific impact of HT pneumatization on enlarging the IAC-JB distance, both in the superior and lateral walls. In the context of pneumatization types affecting the IAC-JB distance, the data reveals that, within the superior wall, the sequence from DPC through NP to HT reflects an increasing trend in this distance. Specifically, the transition from NP to DPC is characterized by a mean decrease of 4.82, indicating that DPC exhibits a decreased distance compared to NP when considering the negative sign. Conversely, HT demonstrates a significant elevation in the IAC-JB distance with a mean difference of 5.765 when compared to DPC, highlighted by its positive value and supported by a statistically significant p-value of less than 0.001. This pattern underscores a progressive enlargement in the IAC-JB distance as one moves from the moderate pneumatization represented by DPC, through the absence of pneumatization (NP) to extensive pneumatization as seen with HT, so the correct sequence from the shortest to the longest distance is DPC, followed by NP and lastly HT.

### Limitations in CBCT visualization of the jugular bulb

CBCT, along with CT and HRCT, serve as invaluable tools in the detailed assessment of bone structures [40]. These imaging techniques offer precise visualization of osseous formations, enabling clinicians and researchers to presume the presence of blood vessels and nerves based on the observable grooves or canals within the bone. However, it’s critical to note the inherent limitation of these methods in directly visualizing soft tissue structures, such as the JB. Several studies have assumed to evaluate the JB using CBCT [18, 39], CT [35], or HRCT [46], yet, it is imperative to distinguish between the direct visualization of the venous structure of the JB itself and the osseous housing or jugular fossa that contains the JB, which is indeed discernible via these imaging methods. A failure to clearly define what is being assessed—whether it is the JB venous structure or its bony encasement—can lead to misinterpretations and inaccuracies within the research. Hence, clarity in the methodological section of such studies is not just beneficial but necessary to ensure the validity of their findings and to avoid disseminating incorrect conclusions about the capabilities of CBCT in evaluating the JB.

### Study limitations

Our study faces some limitations that warrant consideration for future research. Primarily, the dimension of our sample size suggests that studies including a larger number of cases would provide a more robust understanding. Additionally, there is a pressing need for a universally adopted classification system for JB pneumatization and its related anatomical variations. Such a system would facilitate clearer comparisons across studies. Furthermore, the establishment of a standardized approach to addressing anatomical variations, including hypoplasia or hyperplasia, HJBs and JBD, is imperative. This standardization would ensure consistency in diagnosis, treatment planning, and surgical procedures, enhancing patient care outcomes. The acknowledgment and addressal of these limitations are crucial steps toward advancing our understanding and management of JB anatomy and its anomalies.

## Conclusion

We advocate for a collaborative effort to establish standardized classifications of JB walls’ pneumatization and anatomical variations, grounded in evidence-based research and supported by detailed imaging analysis. This approach will not only clarify the existing ambiguity but also pave the way for more personalized and safer clinical practices. The integration of imaging diagnostics, along with universally accepted classification systems, will significantly advance our understanding and management of JB abnormalities, ultimately leading to improved patient care in otolaryngology and related fields.

## Data Availability

No datasets were generated or analysed during the current study.

## Abbreviations

JB: jugular bulb
IAC: internal acoustic canal
HJB: high jugular bulb
JBD: jugular bulb diverticulum/a
HRCT: high–resolution computed tomography
NP: No pneumatization
PMT: Posteromedial tract
DPC: Deep petrosal cell
ILC: Infralabyrinthine cell
HTC: Hypotympanic cell
HT: Hypotympanum
BOC: Basi-occipital cell
AOC: Accessory occipital cell
